# Spring-Wire Technique as a Cost-Effective Alternative for Volar Rim Fragment Fixation in Distal Radius Fractures

**DOI:** 10.7759/cureus.71463

**Published:** 2024-10-14

**Authors:** Mahmoud Salama, Ehab Alieldin, Essam Awad El-Karef, Mohammad Hasan Ahmad

**Affiliations:** 1 Trauma and Orthopaedic Surgery, Alexandria Faculty of Medicine, Alexandria, EGY; 2 Trauma and Orthopaedics, Manchester University Foundation NHS Trust, Manchester, GBR; 3 Trauma and Orthopaedics, Barts Health NHS Trust, London, GBR

**Keywords:** distal end radius fracture, spring wires, volar lunate dislocation, volar plate, volar rim fragment

## Abstract

Introduction

Volar rim fragment fixation is difficult to manage, as it is distal to the watershed line, rendering normal volar plates unable to securely capture it. This fragment must be precisely addressed as volar carpal subluxation is unavoidable when fixation is not efficient. The spring-wire technique maintains a stable fixation of this key fragment, which has been previously described in a small series.

Materials and methods

This is a prospective study of 20 patients who presented to El-Hadara University Hospital in Alexandria between the years 2020 and 2022, with complex distal radius fractures and small volar rim fragments, who were treated by open reduction and internal fixation using the spring-wire technique.

The study was approved by the institutional review board of Alexandria Main University Hospital, and informed consent was taken from all patients in this study ensuring compliance with ethical standards.

All patients underwent open reduction and internal fixation using the spring-wire technique. The procedure was performed by the same surgical team to ensure consistency. Postoperative radiographs were taken to assess reduction quality and fixation. Postoperative radiographic parameters, range of motion, grip strength and the El-Hadara wrist function scoring system were obtained after one year of follow-up, to evaluate results of fixation.

Results

All patients achieved a functional range of motion and grip strength and showed radiological union of the volar rim fragment with no volar carpal subluxation or escape of the volar rim fragment. The El-Hadara wrist function average score was 18.85±2.66, which represented a good overall result. No complications occurred during follow-up, and none of the patients required removal of the implant.

Conclusion

The spring-wire technique maintains a stable fixation of the volar rim fragment that cannot be held securely by volar plating alone. This method offers a low-profile implant that is readily available and simple to apply. Also, the fragment is stabilized by a dual effect by both the spring wire and the volar plate that act as a buttress to volar displacement.

## Introduction

The lunate facet bears most of the force transmitted from the wrist and shares 46% of the contact area with the radio carpal joints when the wrist is in extension and ulnar deviation [1]. It provides attachment to the short radio carpal ligaments; hence, carpal subluxation occurs if the fixation of the lunate facet is compromised [1,2].

Since the volar rim fragment lies distal to the watershed line, traditional volar plates cannot securely stabilize it. There is always a higher chance of screw penetration into the joint or tendon irritation with attempts to place the plate very distal to be able to fix that fragment [1]. Moreover, the 180 degrees of rotation of the fragment together with scarce subchondral bone add to the difficulties encountered with traditional volar plating techniques [3].

The volar rim fragment often appears in comminuted intra-articular distal radius fractures, though it can also present as an isolated issue. Identifying this fragment pre-operatively is crucial for precise fixation. Several techniques have been described for stabilizing this fragment, including tension band wiring, screw and washer fixation, volar hook plates, and arthroscopic reduction with pinning [4].

In 2014, Moore and Dennison introduced the spring-wire technique in a small series of nine patients. They used a bent Kirschner wire to secure the volar rim fragment beneath a volar locking plate, reporting successful fracture healing and maintained reduction in all cases. This technique employed the standard volar plating approach and utilized readily available Kirschner wires to securely hold the fragment [5].

This study aims to evaluate the outcomes of 20 patients with complex distal radius fractures involving a volar rim fragment, treated with open reduction and internal fixation using the spring-wire technique.

## Materials and methods

This is a prospective study conducted at El-Hadara University Hospital in Alexandria from 2020 to 2022. The study was designed to evaluate the clinical outcomes of patients with complex distal radius fractures involving small volar rim fragments, treated using the spring-wire technique.

Twenty patients presented to El-Hadara University Hospital in Alexandria with complex distal radius fractures and small volar rim fragments that were treated by the spring-wire technique fixation. We included all patients with complex distal radius fractures with a small volar rim fragment on X-ray and confirmed on CT < 15 mm, and patients aged from 18 to 50 years. We excluded patients with open and osteoporotic fractures. The patient follow-up period was one year. The study duration was two years from 2020 to 2022.

The study was approved by the Institutional Review Board (IRB) of Alexandria Main University Hospital. Informed consent was obtained from all participants before enrollment in the study, ensuring compliance with ethical standards.

In the operating theatre, the patient was placed supine on the operating table with a radiolucent hand table. A well-padded tourniquet was placed on the upper arm and then it was prepared and draped, a fluoroscopy machine (C-arm) was utilized during the procedure. A standard volar flexor carpi radialis (FCR) approach was used through the interval between the FCR tendon and the radial artery [6].

Blunt dissection was advanced between the flexor pollicis longus (FPL) and the radial artery to expose the pronator quadratus muscle. This muscle was stripped off with the periosteum from the volar surface of the radius by an L-shaped incision reaching to the most ulnar side of the radius, the horizontal limb was placed at the watershed line proximal to the joint line and the vertical limb on its radial border.

**Figure 1:**
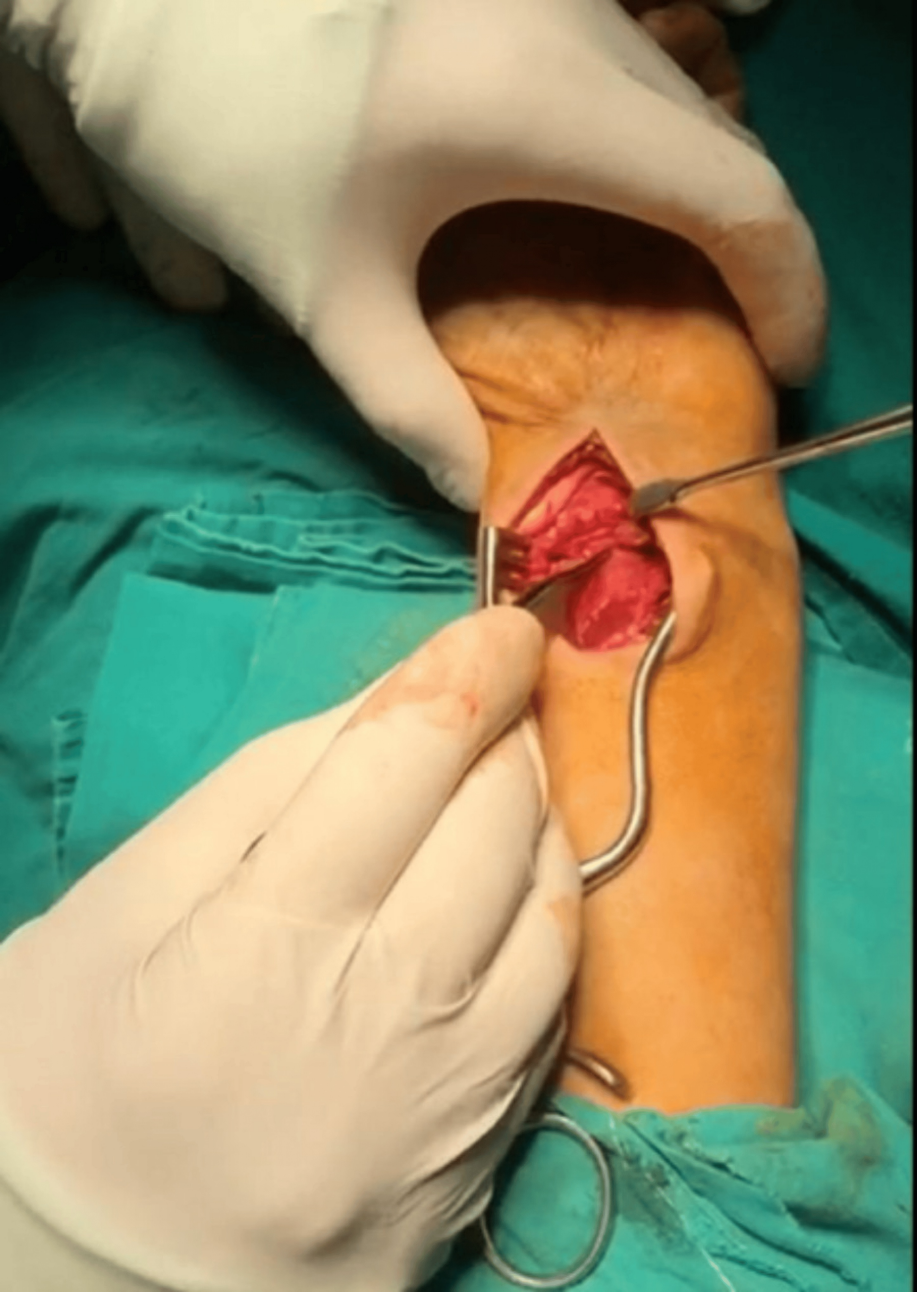
Intra-operative picture of the volar approach and the volar lip fragment

The fracture was then exposed, and reconstruction of the articular surface began with the volar rim fragment. The fragment was manipulated by a dental pick and reduced to its anatomical position. The volar rim fragment in this study was too distal and small to be held stable and secure by two distal screws from the volar plate. One or two small K-wires (0.8 or 1 mm) according to the size of the fragment were placed into the distal volar lip during wrist extension and ulnar deviation to give better access for the K-wires insertion. The K-wires are angled to the opposite intact proximal dorsal cortex and directed radially (Figure 1).

**Figure 2:**
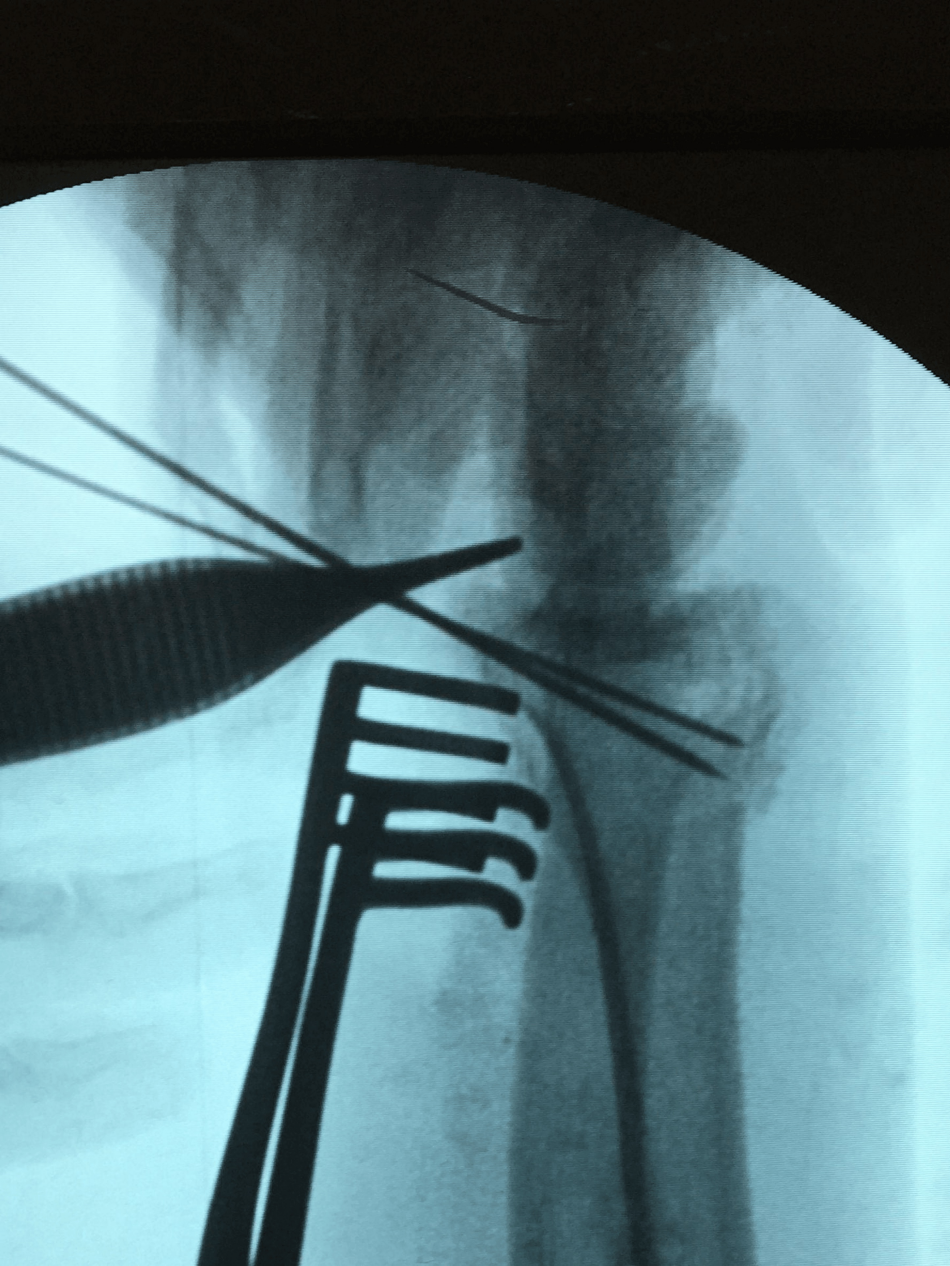
Mobile image intensifier (II) image for the spring-wire technique

**Figure 3:**
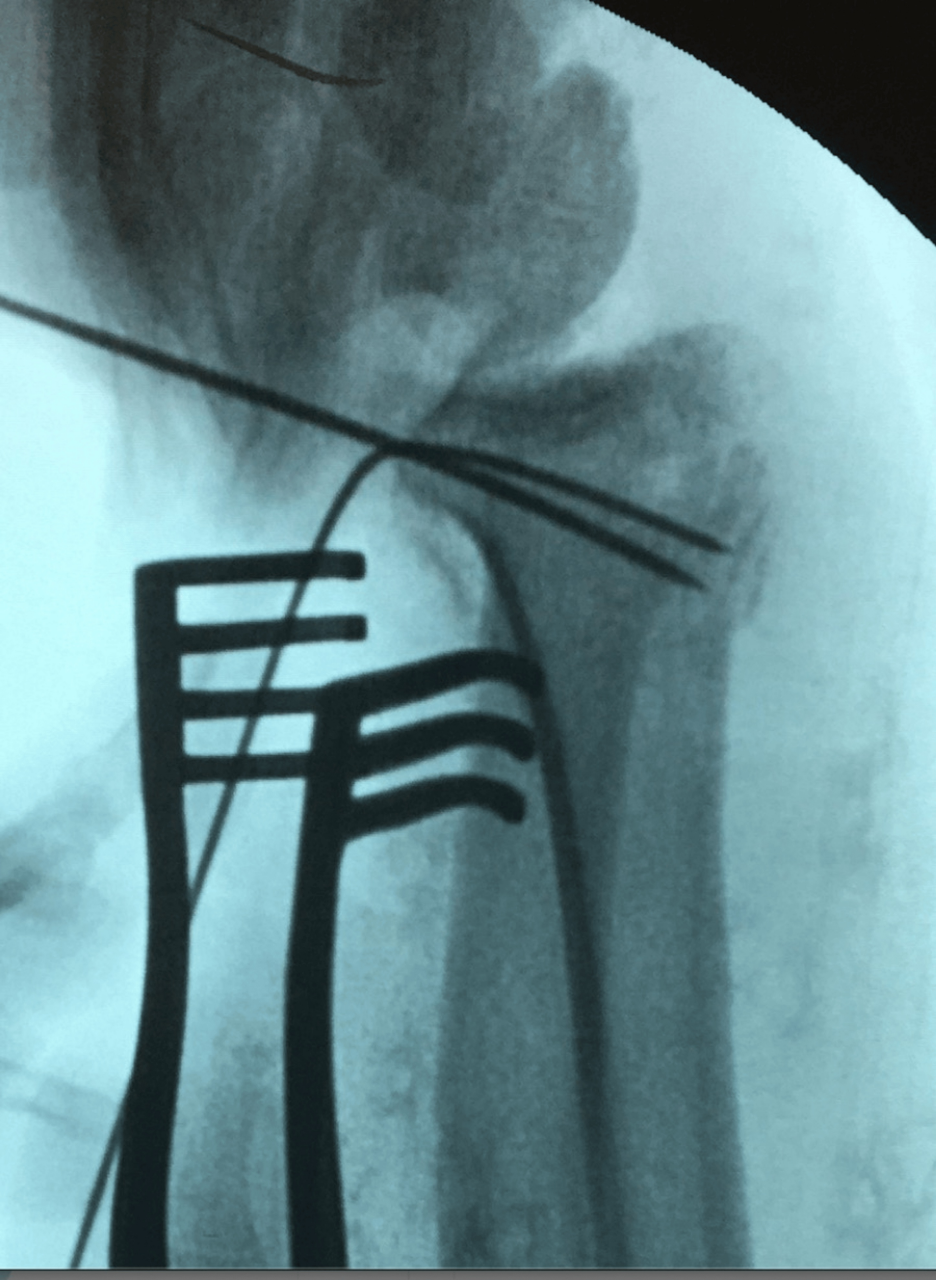
Mobile (II) image for the spring-wire technique

**Figure 4:**
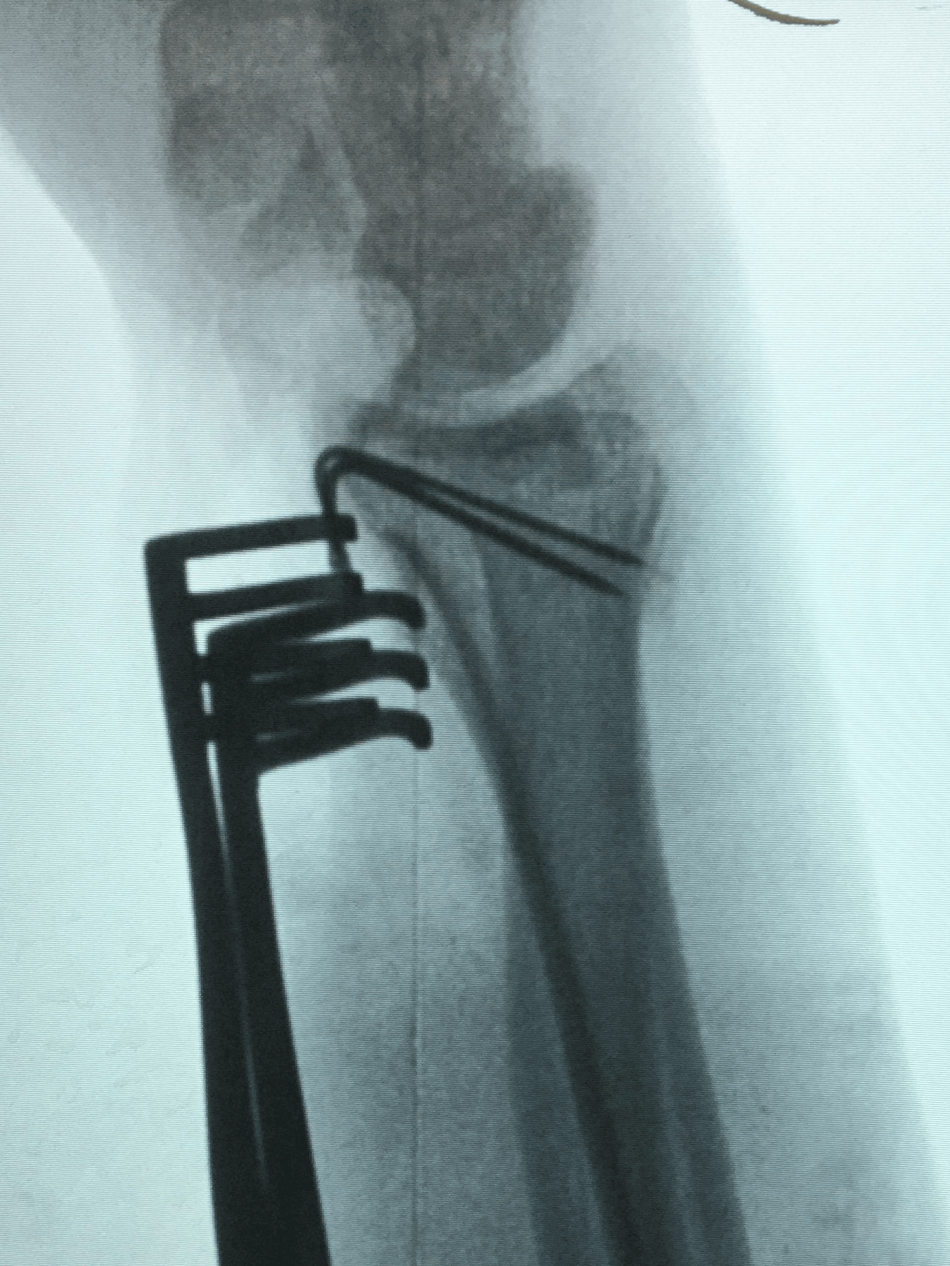
Mobile (II) image for the spring-wire technique

Fluoroscopy was used to evaluate the reduction of the fragment, aiming for a teardrop angle of approximately 70°. Once the reduction was confirmed, the K-wires were carefully advanced into the intact dorsal cortex of the proximal radius using an oscillating technique to minimize the risk of soft tissue injury, particularly to the median nerve. The K-wires were then retracted about 10 mm and gently bent around the volar edge of the radius, forming the spring-wire construct. They were then tapped back into position. The K-wires were left long enough to be secured beneath the volar plate that was applied afterwards (Figures 2-4).

**Figure 5:**
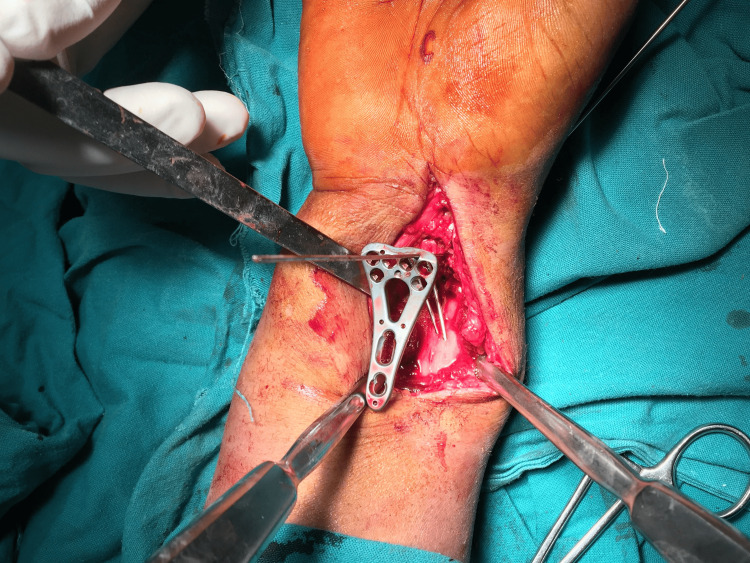
Intra-operative picture of plate position with bent K-wires underneath

**Figure 6:**
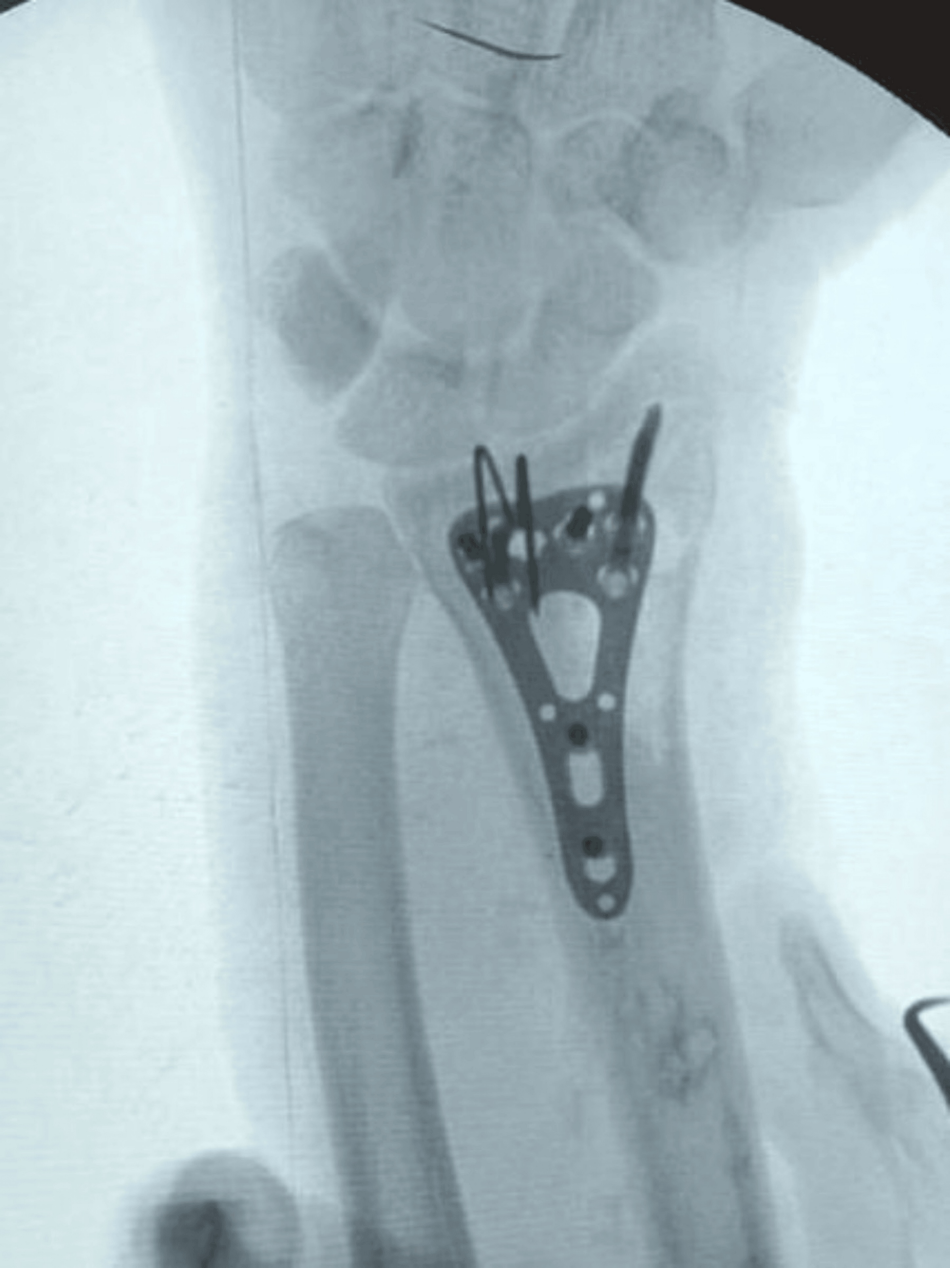
Mobile (II) images of final plate position and spring wires

**Figure 7:**
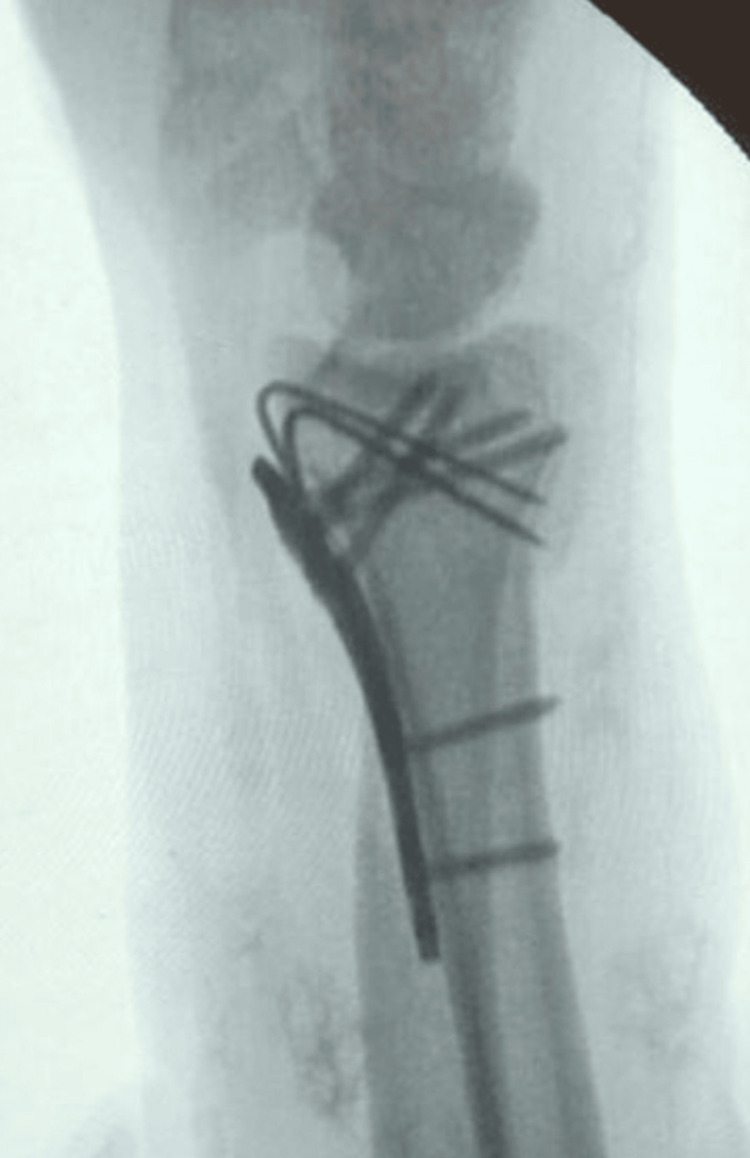
Mobile (II) images of final plate position and spring wires

After reconstructing the intermediate column, the radial column was then addressed and provisionally fixed using K-wires. At this stage, the articular surface was reduced, and the volar plate was applied, covering the spring wires on the radial shaft. A non-locking screw was used to compress the plate to the radial shaft, ensuring not to over-reduce the volar rim fragment and force it into an extended position, which could decrease the teardrop angle. The bent K-wires, sized 0.8-1 mm, were chosen to prevent increasing the gap to the volar cortex beneath the plate and to reduce the risk of irritation to the flexor tendons (Figures 5-7). The articular surface was then secured with distal screws from the volar plate. The stability of the fracture reduction was confirmed through fluoroscopy and physical examination, by palpating the fragment and checking stability by flexion and the extension load applied. A pronated oblique lateral view was used to verify that the K-wires did not protrude beyond the dorsal cortex, minimising the risk of injury to the extensor tendons.

Post-operative evaluation and management

A post-operative below-elbow plaster cast was applied for three weeks until sutures were removed and finger motion was started immediately. After three weeks, the cast was removed and the patient was instructed to start a central arc of wrist and forearm motion (45 degrees) in all directions: flexion, extension, supination and pronation till six-week post-operative and fracture union was confirmed. Physiotherapy was started after confirmation of fracture union to achieve maximum range of motion and grip strength. Patient follow-up with standard posteroanterior and lateral radiographs was done at the following intervals: two weeks, six weeks, three months and one year. Radiographs were used to check fracture reduction, escape of the volar rim fragment, articular congruity, confirming fracture union and development of any complication.

Final clinical assessment was done for all patients at the end of the follow-up period after completing their rehabilitation physiotherapy program. The range of movement was measured with a goniometer. The following movements were measured: wrist flexion and extension, forearm supination and pronation. Grip strength was measured with a Sammons Preston Jamar dynamometer (Sammons Preston Rolyan, Bolingbrook, IL, USA). Any complication was documented.

All clinical findings were evaluated and given scores at the end of the follow-up period of one year according to the El-Hadara wrist function scoring system (Tables 1, 2) [7]. This scoring system was developed by one of our co-authors (Mr Essam Awad El-Karef) and was used to assess wrist function. Permission to use and publish this scoring system was obtained.

**Table 1:** El-Hadara wrist function scoring system

	El-Hadara wrist function scoring system	Point	
Subjective	I.	Patient's satisfaction:	
evaluation:		● Pleased	4
		● Satisfied	3
		● Moderate satisfied	2
		● Dissatisfied	1
	II.	Pain:	
		● No pain	4
		● Mild (occasional at extremes of motion and not interfering with function)	3
		● Moderate (during activities requiring forceful grip)	2
		● Severe (during activities of daily living or during rest)	1
	III.	Sense of stiffness:	
		● Supple	4
		● Mild (occasional)	3
		● Mild (persistent)	2
		● Stiff	1
	IV.	Work capacity:	
		● Return to regular sports/activities	4
		● Modified regular sports/activities	3
		● Has to change job	2
		● Unable to work	1
Objective	I.	Wrist movement:	
evaluation:		● At least 80% of that of the uninvolved hand	4
		● 65 to 79 per cent	3
		● 40 to 64 per cent	2
		● Less than 40 per cent	1
	II.	Grip strength:	
		● At least 80% of that of the uninvolved hand	4
		● 65 to 79 per cent	3
		● 40 to 64 per cent	2
		● Less than 40 per cent	1

**Table 2:** El-Hadara wrist function scoring system

End result point range	Points
Excellent	21-24
Good	17-20
Fair	13-16
Poor	≤12

Statistical analysis

The statistical analysis in the document utilised several methods to evaluate the data. The arithmetic mean was calculated to find the average values while the standard deviation provided insights into the data’s dispersion. The student’s t-test was employed to compare the means of two independent groups; additionally, the chi-square test was used to assess the association between categorical variables. Correlation coefficients were used to determine the strength and direction of the relationship between two variables. Statistical significance was judged at a 5% level (p ≤ 0.05).

## Results

**Figure 8:**
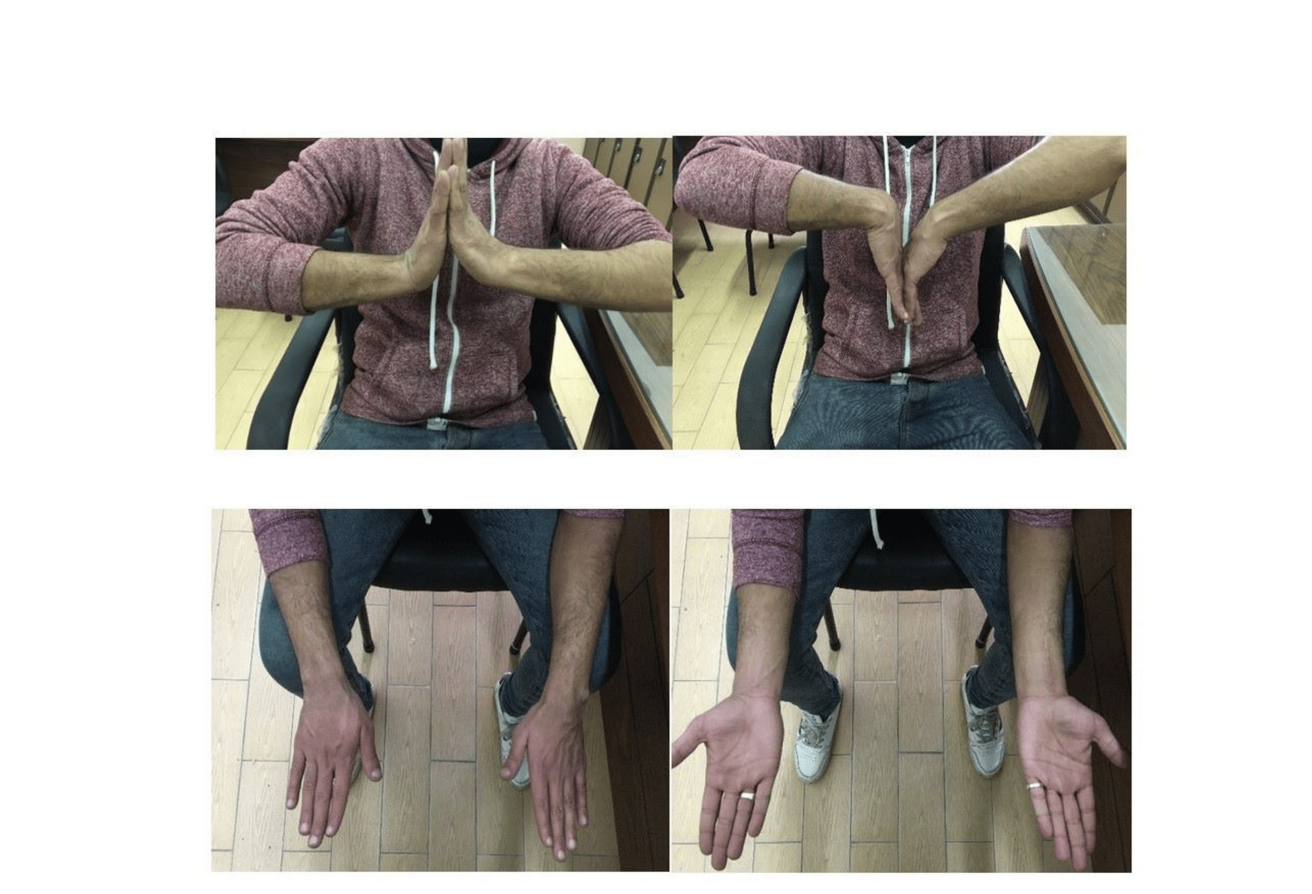
Patient's final range of movement in flexion, extension, pronation and supination

**Figure 9:**
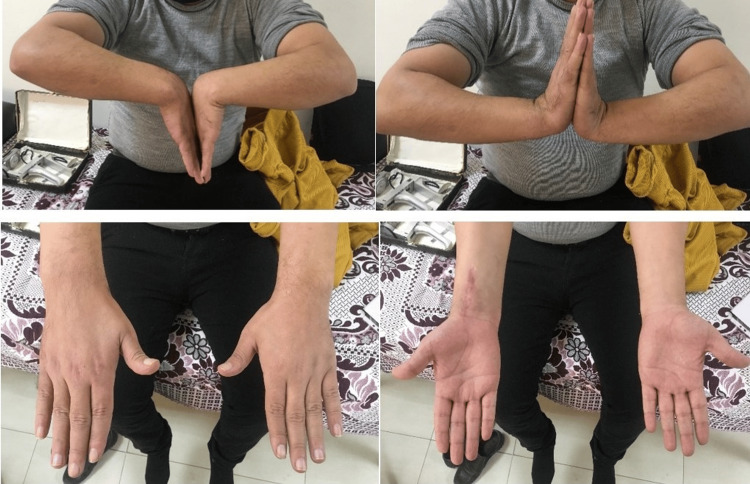
Patient's final range of movement in flexion, extension, pronation and supination

At the end of the one-year follow-up period, patients were assessed by the El-Hadara wrist function scoring system out of 24 points. Results showed that 7 patients (35%) had excellent results (scores between 21 and 23), 10 patients (50%) had good results (scores between 17 and 19) and 3 patients (15%) had fair results (scores between 14 and 16). No patients were graded as having poor results. Results showed that the mean score was 18.85±2.66 (Table 3) (Figures 8, 9).

**Table 3:** Distribution of the studied cases according to the El-Hadara wrist function score (n = 20) No.: Number; %: Percentage

Score	No.	%
Fair	3	15
Good	10	50
Excellent	7	35
Min. – Max.	14-23	
Mean ± SD	18.85±2.66	
Median	18	

Radiological parameters preoperatively were also assessed and correlated to the final outcome, including radial height, radial inclination, volar tilt and ulnar variance. There was no statistical significance between all of the parameters and the final score (Table 4).

**Table 4:** Radiological parameters at different periods of follow-up

Parameter	Pre-operative (Mean ± SD)	Post-operative (Mean ± SD)	1 Year Post-operative (Mean ± SD)
Radial Height (mm)	5.1 ± 2.49	11.2 ± 0.77	11.05 ± 0.99
Radial Inclination (`°)	13.25 ± 9.35	20.0 ± 0.86	20.0 ± 1.83
Volar Tilt (`°)	-7.0 ± 18.38	5.95 ± 5.53	6.50 ± 6.39
Ulnar Variance (mm)	0.85 ± 0.93	-0.3 ± 0.66	-0.25 ± 0.64

 There were no associated complications in the studied patients during the one-year follow-up.

## Discussion

In this study, eight patients (40%) presented with preoperative volar carpal subluxation, highlighting the importance of securing stable fixation of the volar rim fragment to prevent subsequent volar carpal subluxation [8,9].

Our findings align with those of Moore et al. [5], demonstrating that the spring-wire technique provides reliable fixation for small volar rim fragments in distal radius fractures. Our study, conducted on a larger scale, supports the effectiveness of this technique. In all cases, the volar rim fragment united stably, with no cases of post-operative volar carpal subluxation or fragment displacement. All patients achieved a functional range of motion and grip strength, with minimal disability and pain reported. Furthermore, there were no complications, and none of the patients required hardware removal.

There is no universally recommended fixation method for all fracture patterns [3]. Tension band wiring with a figure-of-eight configuration, as described by Minato et al. [10], can be useful for thin volar marginal fragments. While this technique is accessible, it can be technically challenging when combined with volar plating, and K-wires may loosen over time [10]. Using a single cannulated screw with a washer provides a low-profile buttress for the volar rim fragment but lacks rotational stability [11].

The volar hook plate, as a fragment-specific fixation method, has demonstrated favourable results in fracture healing and stability. O’Shaughnessy et al. reported success in a series of 26 wrists with volar marginal rim fractures treated using this approach [11]. However, four patients required hook plate removal due to hardware-related symptoms. Additionally, the high cost of this implant may limit its availability [11]. Arthroscopic reduction and pinning, though effective, are not universally available and require a steep learning curve [12].

The spring-wire technique offers a straightforward and cost-effective solution for stabilizing small volar rim fragments, combining the benefits of spring-wire fixation and volar plating. This method uses a low-profile implant, readily accessible and simple for most surgeons to apply [13]. Importantly, the use of small K-wires minimizes disruption to critical volar carpal ligaments. Additionally, the dual action of the spring wire and the volar plate provides a buttressing effect, preventing volar displacement and ensuring the K-wires do not back out [5].

One limitation of our study is the small sample size, and another is the relatively short follow-up period. However, a one-year follow-up is typically sufficient to observe key outcomes such as loss of reduction or hardware failure [14]. Future research comparing various techniques for stabilizing the volar rim fragment would be beneficial.

This study presents evidence supporting the use of a cost-effective technique involving only K-wires and a volar plate for the fixation of distal radius volar rim fragments. By employing basic instrumentation, this approach could be a viable option for healthcare systems with limited resources such as in certain developing countries, ensuring adequate fixation while avoiding the financial and logistical challenges encountered with more advanced and costly implants.

## Conclusions

In conclusion, the spring-wire technique is a reliable method for the fixation of volar rim fractures, which are difficult to securely fix with conventional volar plates. It is a cost-effective alternative to fragment-specific implants and is easily used by most surgeons. The surgeon is able to address a small volar rim fragment if not identified on preoperative imaging with only a standard volar plate available. Its adoption in clinical practice can potentially improve the management and prognosis of these challenging fractures.
